# Essential function of the integrator complex in Kaposi’s sarcoma-associated herpesvirus lytic replication

**DOI:** 10.1128/jvi.00266-25

**Published:** 2025-08-13

**Authors:** Amy Nguyen, Tianqi Li, Conner Traugot, Kimberly Paulsen, Tiffany S. Nelson, Mingyi Xie, Zhe Ma

**Affiliations:** 1 Department of Molecular Genetics and Microbiology, College of Medicine, University of Florida 3463, Gainesville, Florida, USA; 2 Department of Biochemistry and Molecular Biology, College of Medicine, University of Florida 3463, Gainesville, Florida, USA; 3 UF Health Cancer Center 27509, Gainesville, Florida, USA; 4 UF Genetics Institute, University of Florida 3463, Gainesville, Florida, USA; University of Toronto, Toronto, Ontario, Canada

**Keywords:** integrator, Kaposi's sarcoma-associated herpesvirus, herpesviruses, lytic, reactivation, INTS11

## Abstract

The integrator complex (INT) is an essential regulator of RNA biogenesis across evolution. Most current findings describe INT’s function in states of equilibrium, presenting a research gap in INT’s role in dynamic states, such as in infections and cancers. Viruses hijack cellular RNA machinery to transcribe their genes and produce viral progeny, presenting a unique condition to investigate INT-dependent RNA regulation under perturbation. Kaposi’s sarcoma-associated herpesvirus (KSHV) is an oncogenic DNA virus that causes two deadly cancers, Kaposi’s sarcoma and primary effusion lymphoma. KSHV undergoes a highly regulated and robust transcription of viral genes upon lytic reactivation, providing a complex and dynamic system to investigate integrator-mediated viral/host RNA regulation. We find that integrator subunit 11 (INTS11), the enzymatic core of INT, is essential for KSHV lytic replication triggered by reactivation or primary infection. Further RNA-seq analyses revealed a dynamic and unique signature of human transcriptomes during each lytic stage, respectively. Although the knockdown of INTS11 resulted in selective upregulation and downregulation of certain human gene transcription, INTS11’s loss globally repressed the KSHV transcriptome throughout KSHV lytic replication. This inhibited viral lytic gene expression, viral genome replication, and virion production. Integrator subunits 9 and 6 are also important for KSHV lytic replication. Mechanistically, ChIP-seq analysis showed that INTS11 is increasingly recruited to the KSHV genome with some unique binding patterns as the lytic cycle progresses, suggesting that KSHV hijacks INTS11 during lytic gene transcriptions. In all, our findings reveal the essential roles of the Integrator complex in KSHV lytic replication.

**IMPORTANCE:**

The integrator complex (INT) is essential for RNA metabolism and is fundamental to all organisms, but its function and regulation during viral infection are not well described. Kaposi’s sarcoma-associated herpesvirus (KSHV) infection establishes lifelong infection and causes two deadly cancers; however, no vaccine is available. Using KSHV as a model, we found that integrator subunit 11 (INTS11), the enzymatic core of INT, is recruited to the KSHV genome under lytic phases and plays an essential role in facilitating global KSHV lytic mRNA transcription and viral production. This reveals the critical role of INT in viral infection, a common and inevitable event in human life.

## INTRODUCTION

In metazoans, the integrator complex (INT) is emerging as an essential component in RNA metabolism across evolution (1–3). INT is a multi-functional, 15-subunit protein complex specific to the animal kingdom (1). The complex is known to associate with the C-terminal domain of RNA polymerase II (RNA Pol II), regulate mRNA transcription, and affect RNA Pol II transcription elongation at promoter-proximal sites (4–6). Individual integrator subunits have biochemical distinctions and varying roles (7). As the core of INT, integrator subunit 11 (INTS11) contains endonuclease activity that can process the 3’-ends of small nuclear RNAs (snRNA) and enhancer RNAs (eRNA), both critical components in gene regulation (2, 8). Other INT subunits are involved in complex scaffolding, connecting with the sensor of the single-stranded DNA (SOSS) complex, and the serine/threonine-protein phosphatase 2A (PP2A) (6). The altered expression of INTSs is linked to a variety of cancers, neurodegeneration, and developmental delays, highlighting its prominent role in maintaining proper RNA metabolism (9–13). Although novel functions of the integrator complex continue to emerge, its role in viral infection is less understood.

Viruses are known to hijack cellular machinery to establish infections and produce progeny, providing a dynamic system to investigate RNA regulation. Due to the broad distribution of viruses, exposure to viral infection is inevitable in human life. Thus, RNA metabolism during viral infection is highly physiologically relevant to human health. Surprisingly, despite INT’s critical role in human transcriptional machinery, there are a few reports exploring the function of the integrator complex in the context of viral infection. Xie et al. reported that INT processes the 3’ ends of Herpesvirus saimiri (HVS) microRNAs (miRNAs), leading to their maturation (14). This is the only report of the INT function in virus-infected cells, in a non-human host. Thus, there still exists a research gap in human and viral RNA metabolism under infection.

Kaposi’s sarcoma-associated herpesvirus (KSHV) is a double-stranded (ds)DNA oncogenic gammaherpesvirus known to cause multiple human malignancies, such as Kaposi’s sarcoma, primary effusion lymphoma, and multicentric Castleman’s disease (15–17). Its large 160 kb genome directs the production of more than 90 viral proteins, 25 viral miRNAs, and several long non-coding RNAs (lncRNA) (18, 19). As a herpesvirus, KSHV has a biphasic lifecycle including latent and lytic phases, each phase having unique transcriptional profiles (20). During latency, only four viral latent mRNAs are transcribed (20–22). In the lytic phase, viral lytic mRNAs are actively transcribed throughout the immediate early (IE), early (E), and late (L) lytic stages (20, 23, 24). KSHV facilitates these transcriptional activities by recruiting RNA Pol II to its genome (25, 26). For example, KSHV ORF57 has been shown to trap RNA Pol II in specific regions on the KSHV genomes and facilitate the initiation of viral lytic replication (27, 28). It is widely reported that RNA Pol II is strongly associated with INT (2, 5). Given KSHV’s dependence on RNA Pol II to exhibit diverse transcriptional activities, KSHV infection can provide a complex dynamic system to investigate the role and regulatory mechanisms of the Integrator complex during viral infection.

Here, we report that integrator subunit 11 (INTS11) is essential for optimal KSHV lytic replication. In our iSLK.219-based latent to lytic reactivation model, the knockdown of INTS11 leads to the abolition of KSHV lytic gene transcription and translation, which ultimately inhibits KSHV genome replication and viral propagation. Similar phenotypes are observed in our primary infection to the lytic model, where a deficiency of INTS11 attenuates the production of KSHV lytic mRNAs and proteins. In contrast, INTS11 plays a minor role in latency following primary infection. Other INT subunits, INTS9 and INTS6, also facilitate the KSHV lytic replication. Ribosomal RNA-depletion RNA sequencing (ribo-depletion RNA-seq) analyses reveal distinct and dynamic regulatory patterns by INTS11 in the human and viral transcriptome, through different stages of a KSHV lytic cycle. Although human transcription is diversely regulated, nearly all viral transcription is significantly blocked upon INTS11 knockdown. Moreover, transitioning from the latent to the late lytic phase, the number of INTS11-dependent human genes drops from 30% to 11% of the total human genes. This is consistent with our chromatin-immunoprecipitation sequencing (ChIP-seq) data performed in iSLK.219 cells, which shows that at 72 h after lytic reactivation, INTS11 is increasingly recruited to the whole KSHV genome, but less INTS11 recruitment is detected in the human genome. INTS11 ChIP-seq signals peak in two hotspots on the KSHV genome, which could be crucial for the role of the integrator complex during viral infection. Collectively, our results suggest that the integrator complex is recruited to the KSHV genome during the KSHV lytic phase, and this dynamic process is essential for optimal KSHV lytic replication.

## MATERIALS AND METHODS

### Cell lines

SLK, iSLK.RTA, and iSLK.219 (29) cells were obtained from the Rolf Renne lab at the University of Florida. The cells were maintained in DMEM (Corning, Cat#10-013-CV) supplemented with 10% FBS (Gibco, 10437-028), 1% penicillin, and streptomycin (Corning, Cat#30-001-CL). For iSLK.RTA, G418 (250 µg/mL) (Invivogen, Cat# ant-gn-5) and puromycin (10 µg/mL) (Invivogen, Cat# ant-pr-5b) were used. For iSLK.219 cells, G418 (250 µg/mL), hygromycin (400 µg/mL) (Invivogen, Cat# ant-hg-5), and puromycin (10 µg/mL) were used. All cell lines were kept in an incubator at 5% CO_2_ and 37°C.

### siRNA transfection

Transfection of siRNA synthesized from IDT DNA was done using the TransIT-X2 Dynamic Delivery System (Cat# MIR 6004) following the manufacturer’s protocol. Sequences are as follows (where NS represents nonspecific and * represents siRNA-resistant strand):

siNS 5′-phos/AAGCGAUACCUCGUGUGUGAdTdT-3′

siNS* 5′-UCACACACGAGGUAUCGCUUdTdT-3′

siINTS11 5′-phos/UCGAAGGCCUUGAUGUGCUdTdT-3′

siINTS11* 5′-AGCACAUCAAGGCCUUCGAdTdT-3′

siINTS9 5′-phos/UACUAAGGGCAGAGUUCACdTdT-3′

siINTS9* 5′-GUGAACUCUGCCCUUAGUAdTdT-3′

siINTS6 5′-phos/AAUGCAUCAAUUUCUUUCCdTdT-3′

siINTS6* 5′-GGAAAGAAAUUGAUGCAUUdTdT-3′

### The SLK, iSLK.RTA, and iSLK.219 system

The iSLK.RTA cell line, derived from SLK, contains a doxycycline (Dox)-inducible RTA transgene, a necessary and sufficient protein for KSHV lytic reactivation (29). The iSLK.219 cell line contains the rKSHV.219 genome, which has a dual reporter element expressing green fluorescent protein (GFP) under the control of a constitutively active EF-1 promoter, indicative of the KSHV latent phase. Dox-induced RTA will also bind to the PAN promoter and stimulate red fluorescent protein (RFP) expression as an indicator of KSHV lytic phase entry. At 0, 24, 48, and 72 h post-Dox induction, GFP- and RFP-positive cells were imaged at an exposure of 0.1 s at 4× magnification using the EVOS M5000 microscope. GFP and RFP fluorescence intensity were quantified using the Synergy H1 microplate reader. Mean relative fluorescence values taken from 21 points in each well were graphed as an RFP/GFP ratio to assess levels of lytic reactivation per KSHV-infected cell.

### Real-time quantitative PCR

RNA and DNA were extracted from treated cells using the QIAGEN RNeasy Plus Mini Kit (Cat #74004) or QIAGEN DNeasy Blood and Tissue Kit (Cat#69504). cDNA was synthesized using the RT Master Mix for real-time quantitative PCR (qPCR) II from MedChemExpress (Cat#HY-K0510A). The housekeeping gene β-Actin was used to normalize transcript levels. An equal volume of supernatants was harvested and treated with DNase to remove free KSHV genome DNA, and DNA encapsidated was purified to measure extracellular KSHV genome copies. The viral DNA was quantified using primers specific to the KSHV ORF39 genome sequences. Cellular KSHV genome DNA was quantified using genome β-Actin primers to normalize KSHV intracellular genome copies. The primer sequences used are listed below in Table 1.

**TABLE 1:** Real-time PCR primers

Gene	Forward sequence (5′−3′)	Reverse sequence (5′−3′)
KSHV ORF57	TGGACATTATGAAGGGCATCCTA	CGGGTTCGGACAATTGCT
KSHV ORF39	GTGGGAGTATTCGTGGGTTATC	GGTGAACAGTCGGAGTTCTATC
KSHV K8.1	AAAGCGTCCAGGCCACCACAGA	GGCAGAAATGGCACACGGTTAC
Human INTS11	AGACGGTCCAGGTAGATGAT	CAGACTCTGAGCCCACTTTAAT
Human INTS9	GAGTGCTCGGGTCATGTATTT	GAAGGTAACAGCCTCTGAATGT
Human INTS6	GCCCATCTTACTGTTCCTGATAG	GTTTCTTCCCTGCCCATAGTT
Human Pre-U1	CAGGGCGAGGCTTATCCATTG	GTTTTTGAAACTCCAGAAAGTCAGG
Human β-Actin (RNA and DNA)	AAGACCTGTACGCCAACACA	AGTACTTGCGCTCAGGAGGA
Human GAPDH	GTCTCCTCTGACTTCAACAGCG	ACCACCCTGTTGCTGTAGCCAA

### Antibodies

Anti-human INTS11 (Cat#15860-1-AP) was purchased from Proteintech. Anti-human INTS9 (Cat#13945S) was purchased from Cell Signaling Technology. Anti-INTS6 antibody (Cat#ab86369) was purchased from Abcam. Anti-KSHV LANA (#CatPA0050) was purchased from Novocastra. Anti-KSHV ORF57-HRP (Cat#sc-135746) and anti-human β-Actin-HRP (Cat#sc-47778) were purchased from Santa Cruz. Anti-KSHV ORF45 (Cat#MA5-14769) was obtained from Invitrogen. Mouse anti-viral interleukin-6 (vIL6) antibody (30) is a kind gift from Dr. Blossom Damania.

### KSHV whole transcriptome

The qRT-PCR primer set was designed for each KSHV open reading frame. iSLK.219 cells were treated as described. RNA was extracted from duplicate samples, and KSHV viral transcript levels were analyzed using a KSHV real-time qPCR-based whole genome array. mRNA levels of viral genes were normalized to the mRNA levels of multiple cellular housekeeping genes to yield dCT as a measure of relative expression. These were then subjected to unsupervised clustering. A heat map and dendrogram depicted by the brackets are shown. Higher transcript expression levels are indicated by red, and lower expression levels by blue, as shown in the key.

### KSHV primary infection assay

HEK293, SLK, or iSLK.RTA cells were transfected with siNS and siINTS11 for 48 h. The supernatant containing extracellular KSHV virions from Dox-induced iSLK.219 cells was harvested at 72 h post-reactivation and added in equal amounts to treated SLK cells. Cells were spinoculated for 90 min with polybrene and harvested at 0, 8, 24, and 48 h post-infection for further analysis.

### Ribosomal RNA depletion RNA sequencing

iSLK.219 cells were transfected with siINTS11 for 48 h. Doxycycline was added to induce the iSLK.219 cells into the lytic phase. The cells were collected at 0, 24, 48, and 72 h post-reactivation (hpr). Total RNA was isolated from cells using TRIzol from LifeTech (Cat#15596018) following the manufacturer’s protocol. The libraries were generated using the SMARTer Stranded Total RNA-Seq Kit v3 following the manufacturer’s protocol (Takara Cat#634485). Ribosomal RNA depletion was performed during RNA-seq library preparation to increase the coverage of mRNA in the library.

### Ribo-depletion RNA-seq data analysis

All RNA-seq data were first processed by Trimmomatic to remove adapter sequences and low-quality base calls (31). The forward and reverse paired reads were then combined into one forward read using PEAR (32). PCR-duplicated reads were collapsed using fastx-collapser (33), and unique molecular tags (UMTs) generated during library preparation were removed using Cutadapt (34). Reads were mapped using HISAT2 (35) to both the human reference genome (GRCh38 release 106) and the KSHV reference genome (GQ994935.1) using default parameters. Read counts for each gene in the human and KSHV genomes were generated with Htseq-count (36) using union mode. Differential gene expression analysis was completed using edgeR (37) using the generalized linear model approach to allow for comparisons across multiple time points and knockdown conditions. Volcano plots were generated using the default R plot function. Gene ontology (GO) functional analysis of the differentially expressed genes was completed using the Bioconductor clusterProfiler (38) tool. The top 10 most significantly enriched GO terms for each domain were plotted using ggplot2 (39). Venn diagrams comparing the significantly upregulated and downregulated genes were created using the Venn diagram R package (40).

### Chromatin immunoprecipitation-sequencing (ChIP-seq)

iSLK.219 cells were cross-linked by adding 1% formaldehyde to the media and gently shaken for 10 min at room temperature. Crosslinking was halted by adding glycine at a final concentration of 125 mM. The cells were collected and centrifuged at 4°C, 500 × *g* for 5 min. Cell lysates were prepared by adding 1 mL of sonication buffer (0.1% SDS, 1% Triton X-100, 10 mM Tris-HCl, 1 mM EDTA, 0.1% NaDOC, 0.25% Sarkosyl, 1 mM DTT, and protease inhibitors) per 40 million cells, followed by shearing of DNA to achieve an average fragment size of 100–300 bp. The sonicated samples were centrifuged at 4°C, 12,000 × *g* for 10 min, and the supernatant was collected with 5% saved as input. The samples were then incubated overnight at 4°C with 5 µg antibody/IgG, 40 µL Protein G beads, and protease inhibitors. The immunoprecipitation (IP) was washed with various buffers, including RIPA 0.3 buffer RIPA 0.3 buffer (0.1% SDS, 1% Triton X-100, 10 mM Tris-HCl, 1 mM EDTA, 0.1% NaDOC, 0.3 M NaCl), RIPA 0 buffer (0.1% SDS, 1% Triton X-100, 10 mM Tris-HCl, 1 mM EDTA, 0.1% NaDOC), LiCl buffer (250 mM LiCl, 0.5% NP-40, 0.5% NaDOC, 1 mM EDTA, 10 mM Tris-HCl), and TE buffer (10 mM Tris-HCl, 1 mM EDTA). SDS elution buffer was added to the beads and incubated at 65°C for 6 h with Proteinase K and RNase A. Finally, DNA was purified using Phenol/Chloroform/Isoamyl alcohol. The libraries were generated using the ThruPLEX Tag-seq Kit following the manufacturer’s protocol (Takara #R400584).

### ChIP-seq data analysis

All ChIP-seq data were first processed by Trimmomatic (31) to remove adapter sequences and low-quality base calls. The forward and reverse paired reads were then combined into one forward read using PEAR (32). PCR-duplicated reads were collapsed using fastx-collapser (33), and unique molecular tags (UMTs) generated during library preparation were removed using Cutadapt (34). Reads were mapped using HISAT2 (35) to both the human reference genome (GRCh38 release 106) and the KSHV reference genome (GQ994935.1) using default parameters. ChIP peaks were called using the MACS2 (30) callpeaks function and compared across time points using the bdgdiff function. A normalization factor for INTS11 ChIP reads was calculated by multiplying by a million, dividing by the total number of reads for each sample, then dividing by the percentage of reads in the ChIP input sample that mapped to the KSHV genome, generating a KSHV viral titer-adjusted CPM metric. Bigwig files for visualization of ChIP read location were generated using the bamCoverage function from Deeptools2 (41) with a smooth length of 60 and multiplied by the normalization factor. The average INTS11 read localization across human and KSHV genes was created using the computeMatrix scale-regions function from deeptools2 with the settings -m 5000 -b 1000. Heatmaps and metagene plots of the location of INTS11 ChIP reads across human or KSHV genes were generated using the deeptools2 plotHeatmap and plotProfile functions.

### Statistical analysis

The statistical significance of differences in mRNA levels, viral titers, and fluorescence intensities in the reporter assay was determined using an unpaired Student’s *t*-test. * indicates *P* < 0.05, ** indicates *P* < 0.01, *** indicates *P* < 0.001, **** indicates *P* < 0.0001.

## RESULTS

### INTS11 deficiency causes massive and global repression of KSHV lytic gene transcription

**Fig 1:**
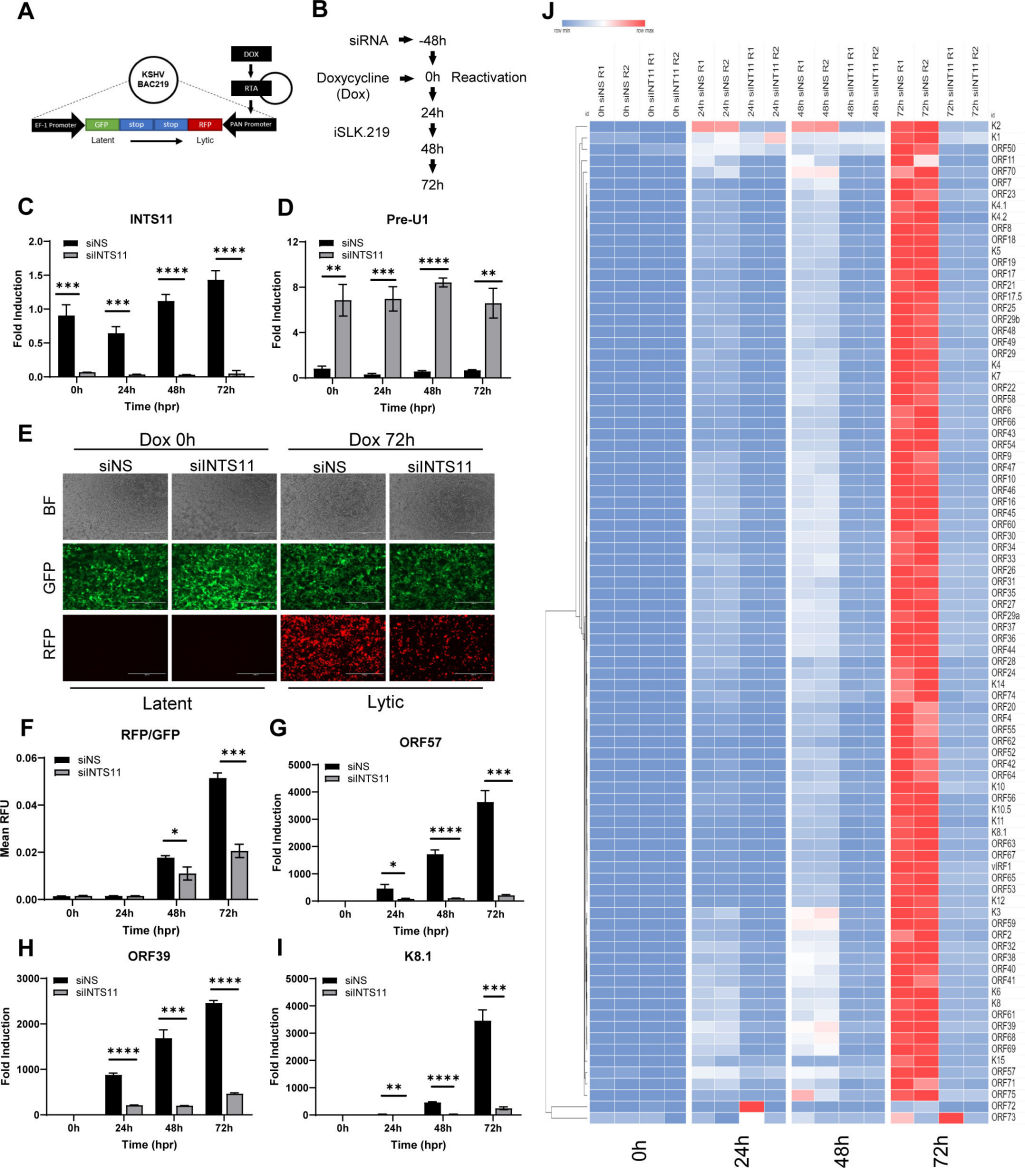
INTS11 deficiency causes massive and global repression of KSHV lytic gene transcription. iSLK.219 cells were transfected with control non-specific (NS) siRNA or INTS11 siRNA for 48 h and then treated with doxycycline (Dox, 0.2 µg/mL). (**A**) iSLK.219 cell construct contains a reporter element constitutively expressing GFP and RFP when treated with Dox. (**B**) Experiment schematic. (**C and D**) RNA was extracted, cDNA was synthesized, and the knockdown efficiency of (**C**) INTS11 and mRNA expression levels of (**D**) Pre-U1 were measured by RT-PCR. (**E**) Treated iSLK.219 cells were imaged at 0, 24, 48, and 72 h post reactivation. (**F**) RFP and GFP fluorescence values were measured using a microplate reader and graphed as a GFP/RFP ratio. mRNA expression levels of KSHV (**G**) ORF57, (**H**) ORF39, and (**I**) K8.1 were measured by RT-PCR. Target mRNA expression was normalized to GAPDH mRNA and presented as fold induction. (J) iSLK.219 cells were transfected with siNS or siINTS11 as described in the text and for panel B. A real-time qPCR-based KSHV transcriptome array was performed. Higher transcript expression levels are indicated by red and lower expression levels by blue as shown in the key. Details about this assay are described in Materials and Methods. hpr = hours post-reactivation. Error bars are representative of the standard deviation from three experiments analyzed with unpaired *t*-test statistics. (*P < 0.05, **P < 0.01, ***P < 0.001, ****P < 0.0001).

INTS11 is essential for cell homeostasis, which makes it challenging to generate CRISPR KO stable cell lines. To investigate the role of INTS11 in the context of KSHV lytic reactivation, we transfected INTS11 siRNA into the iSLK.219 cell line, a valuable *in vitro* system for analyzing changes from KSHV latency to lytic reactivation (Fig. 1A). In total, 48 h post-transfection, doxycycline (Dox) was added to induce the KSHV lytic cycle, and changes were assessed at 0, 24, 48, and 72 h post-reactivation (hpr) (Fig. 1B). INTS11 knockdown efficiency was confirmed at the mRNA level (Fig. 1C). snRNA U1 is a prominent substrate RNA for the Integrator complex. Accordingly, when INTS11 was knocked down, there was an accumulation of misprocessed snRNA U1 (Pre-U1) transcripts (Fig. 1D). The iSLK.219 cell line harbors the whole KSHV genome (rKSHV.219). It also contains a dual reporter element expressing green fluorescent protein (GFP) under the control of a constitutively active EF-1 promoter, indicative of the KSHV latent phase. Upon Dox addition, RTA is induced, which is sufficient to drive the switch from KSHV latency to lytic reactivation. Dox-induced RTA will bind to the PAN promoter and stimulate red fluorescent protein (RFP) expression, indicative of the KSHV lytic phase. Upon lytic reactivation, a significantly reduced RFP signal was observed in siINTS11 cells compared with siNS groups, especially at 72 hpr (Fig. 1E). GFP and RFP fluorescent values were quantified, and significantly fewer RFP/GFP ratios were observed in siINTS11 cells (Fig. 1F). The mRNA levels of representative KSHV lytic immediate early ORF57, early ORF39, and late K8.1 genes were assessed, and the knockdown of INTS11 reduced the mRNA levels of all three representative KSHV lytic genes (Fig. 1G through I) tested. Following the significant inhibitory effects observed on all chosen KSHV ORFs, further evaluation was expanded to the entire KSHV ORF transcriptome. We utilized a modified system as previously described by other groups, but with newly designed primers to specifically target each of the 88 KSHV ORFs (42, 43). The same experiment as described in Fig. 1B was performed, RNA was isolated, and cDNAs of all groups were subjected to an RT-PCR-based KSHV transcriptome array at each time point upon reactivation. Strikingly, INTS11 deficiency caused a massive and global repression of nearly all KSHV-encoded ORF transcripts, highlighting the essential role of INTS11 in maintaining an optimal KSHV lytic cycle (Fig. 1J). Consistently, when INTS11 is knocked down with the same siRNA, we also observed attenuated KSHV lytic gene transcription and translation in BCBL1, a patient-derived primary effusion lymphoma cell line (Fig. S1A through F).

### INTS11 is required for optimal KSHV lytic replication

**Fig 2:**
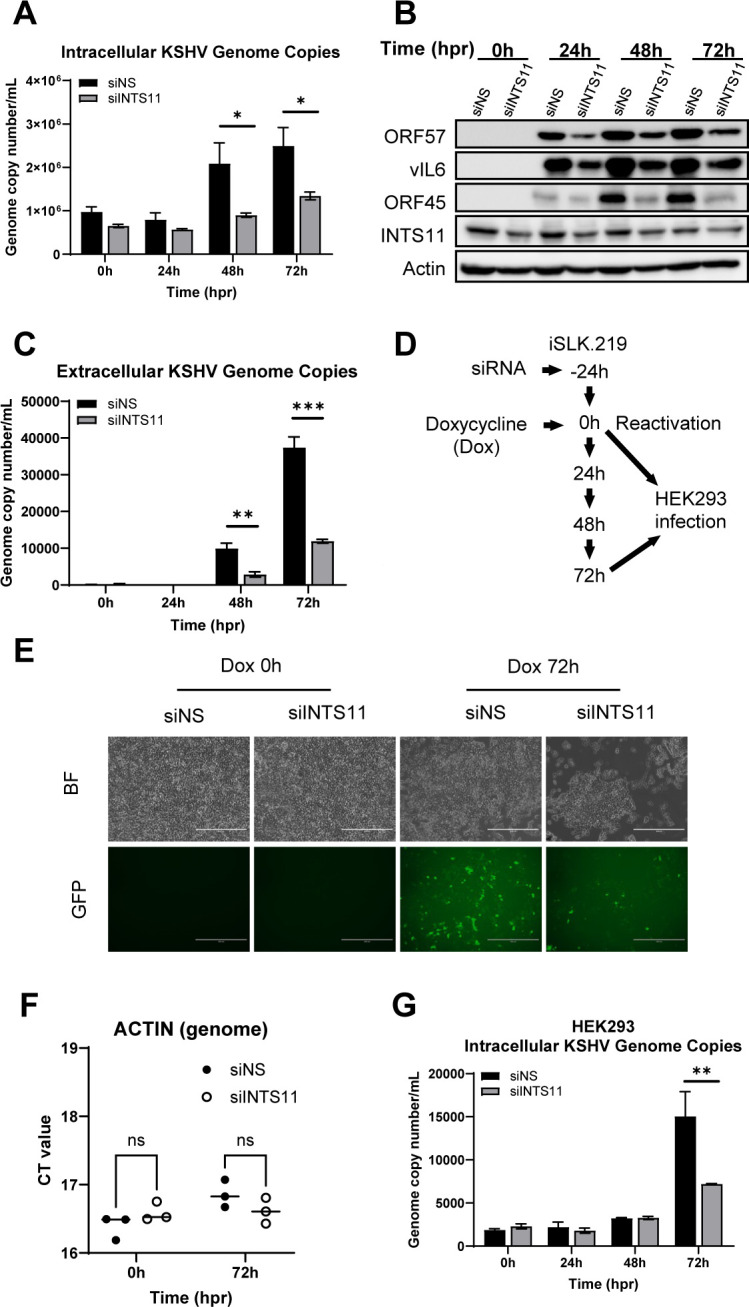
INTS11 is required for optimal KSHV genome replication, protein production, and virion production. Experiments were performed as described in Fig. 1B, (**A**) Genomic DNA was extracted and measured for the number of KSHV ORF39 copies/mL, normalized to genome β-Actin copies with real-time PCR. (**B**) Cell lysates were collected at the indicated time points, and western blots were performed with the indicated antibodies. (**C**) The supernatants were collected from treated iSLK.219 cells, and extracellular virion production was measured using the KSHV ORF39 gene. (**D–G**) Equal volumes of supernatant from 72 h reactivated iSLK.219 cells transfected with siNS or siINTS11 were added to naïve HEK293 cells to assess *de novo* KSHV infection. (**D**) Infection assay experiment schematic. (**E**) KSHV infection was visualized for GFP-positive cells at 24 hpi. (**F**) Ct (cycle threshold) of Actin qPCR in the infected HEK293 cells. (**G**) KSHV genome copy number was measured using the ORF39 genome primers and normalized to genome β-Actin copies with qPCR. hpr = hours post-reactivation. Error bars are representative of the standard deviation from three experiments analyzed with unpaired *t*-test statistics. (*P < 0.05, **P < 0.01, ***P < 0.001).

Given the significant phenotype of INTS11 knockdown on KSHV lytic transcriptome, we next evaluated the impact of INTS11 deficiency on viral lytic cycle progression. Sufficient transcription of KSHV lytic genes is essential for the lytic replication of the KSHV genome, lytic protein expression, and virion production. With insufficient INTS11, significant defects in viral genome replication were observed (Fig. 2A). Select viral protein expression in KSHV ORF57, vIL6, and ORF45 decreased with INTS11 knockdown (Fig. 2B). In addition, knockdown of INTS11 was found to significantly decrease virion production at 48 h and 72 h, as quantified by the extracellular KSHV genome copy number (Fig. 2C). The impact of INTS11 knockdown on infectious virion production was validated by harvesting equal volumes of supernatant from each time point from siRNA-treated iSLK.219 cells and used to infect naïve HEK293 cells as described in Fig. 2D.

At 72 h post-infection (hpi), HEK293 cells, infected by the viruses generated from siINTS11-treated iSLK.219 cells, expressed less GFP and carried fewer KSHV genome copies compared with the siNS group under a similar number of cells (reflected by actin genome) (Fig. 2E through G). Collectively, these data suggest that deficient INTS11 causes reduced viral genome replication, protein expression, and virion production, essential steps during the lytic stage.

### INTS11 is dispensable for the default KSHV primary infection to the latency route

**Fig 3:**
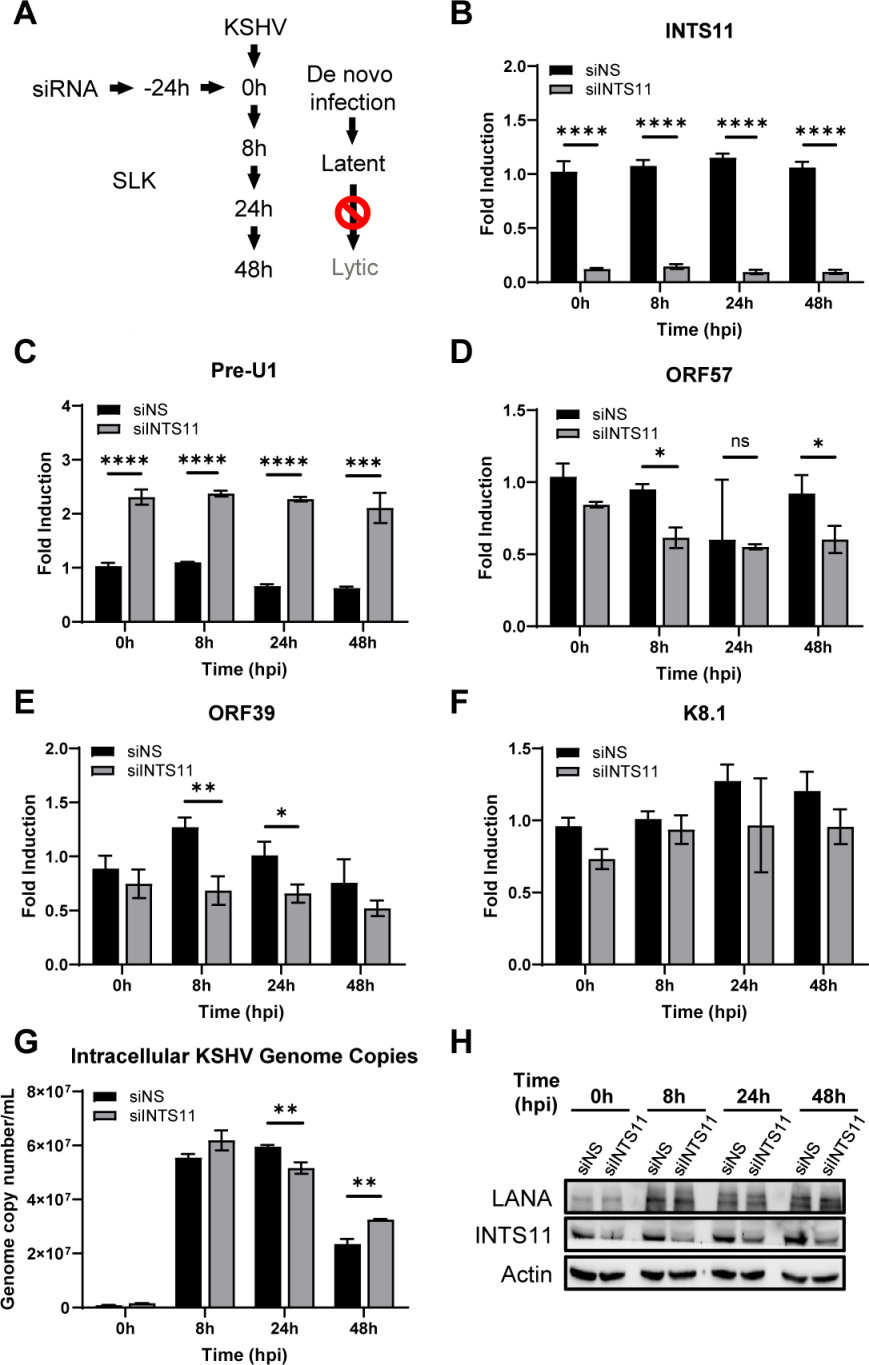
INTS11 is dispensable for the default KSHV primary infection to the latency route. SLK cells were treated with siNS or siINTS11 siRNA for 24 h. Equal volumes of supernatant containing KSHV virions from Dox-treated iSLK.219 cells were harvested and added to siINTS11-treated SLK cells. (**A**) Experiment schematic. (**B–F**) RNA was extracted, and cDNA was synthesized at the indicated time points post-infection. The knockdown efficiency of (**B**) INTS11 and the mRNA expression levels of (**C**) Pre-U1 were measured by real-time PCR. The mRNA expression levels of KSHV genes (**D**) ORF57, (**E**) ORF39, and (**F**) K8.1 were measured using real-time PCR. Target mRNA expression was normalized to GAPDH mRNA and presented as fold induction. (**G**) Genomic DNA was extracted and measured for the number of KSHV ORF39 copies/mL normalized to β-Actin with real-time PCR. (**H**) Cell lysates were collected at the indicated time points, and western blots were performed with the indicated antibodies hpi = hours post-infection. Error bars are representative of the standard deviation from three experiments analyzed with unpaired *t*-test statistics. (*P < 0.05, **P < 0.01, ***P < 0.001, ****P < 0.0001).

The step immediately following virion production is the primary infection of naive cells to expand viral infection in the host. The default phase that the virus enters after primary infection is latency. To investigate whether INTS11 plays a role in assisting latency establishment after KSHV primary infection, INTS11 was knocked down using siRNA in naïve SLK cells and the infection status at 8, 24, or 48 h post-infection was evaluated (Fig. 3A). INTS11 knockdown efficiency and disruption of function were validated at the mRNA level and by the increase of Pre-U1 (Fig. 3B and C). Under the establishment of latency, clear and consistent trends were not observed upon the loss of INTS11. KSHV ORF57, ORF39, or K8.1 mRNA levels were not induced at any time points (Fig. 3C through E), indicating that the cells did not enter the lytic phase. At 8, 24, and 48 h post-infection, INTS11 knockdown led to no differences, a slight decrease, and a mild increase in KSHV genome copies, respectively (Fig. 3G). KSHV genome copy numbers exhibited a decreasing trend as time progressed, suggesting that KSHV was not replicating its genome and it established latency rather than a lytic cycle (Fig. 3G). KSHV latent protein LANA was expressed in the infected cells, representing the successful establishment of latency, but no differences at each time point were observed upon loss of INTS11 (Fig. 3H). These data suggest that INTS11 is dispensable during the default KSHV primary infection to the latency route. Interestingly, when we knock down INTS11 in the pre-established iSLK.219 cells, we also failed to observe repression on latent gene transcription. Surprisingly, K12 was even upregulated (Fig. S2). The detailed mechanisms behind the discrepancy from latent gene to lytic gene transcription need to be further studied.

### INTS11 is necessary for non-canonical KSHV primary infection to the lytic route

**Fig 4:**
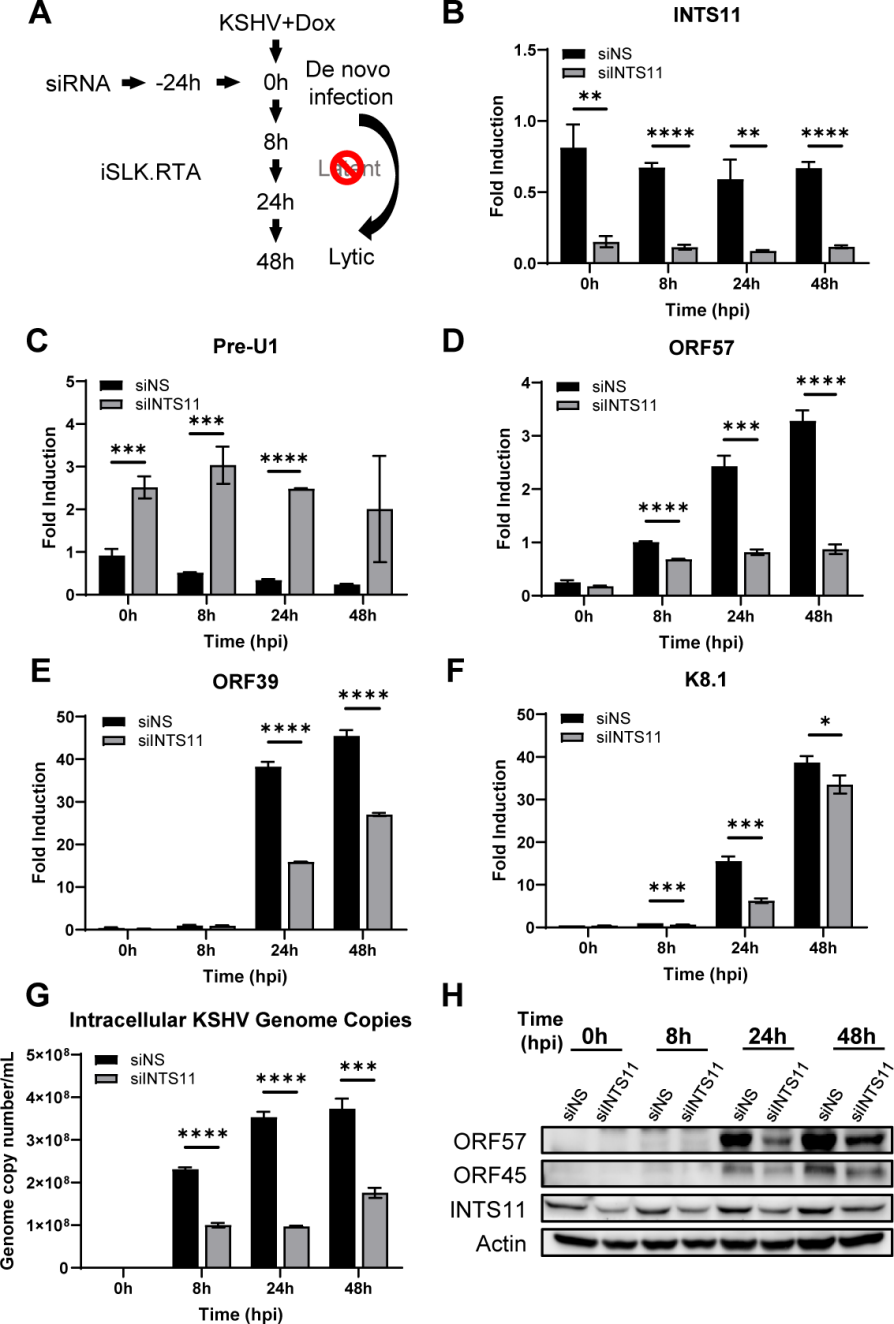
INTS11 is necessary for non-canonical KSHV primary infection to the lytic route. iSLK.RTA cells were treated with siNS or siINTS11 siRNA for 24 h. Equal volumes of supernatant containing KSHV virions from Dox-treated iSLK.219 cells were harvested and added to the treated SLK cells. (**A**) Experiment schematic. (**B–F**) RNA was extracted, and cDNA was synthesized at the indicated time points post-infection. The knockdown efficiency of (**B**) INTS11 and the mRNA expression levels of (**C**) Pre-U1 transcripts were measured by real-time PCR. The mRNA expression levels of KSHV genes (**D**) ORF57, (**E**) ORF39, and (**F**) K8.1 were measured using real-time PCR. Target mRNA expression was normalized to GAPDH mRNA and presented as fold induction. (**G**) Genomic DNA was extracted and measured for the number of KSHV ORF39 copies/mL normalized to β-Actin, with real-time PCR. (**H**) Cell lysates were collected at the indicated time points, and western blots were performed with the indicated antibodies hpi = hours post-infection. Error bars are representative of the standard deviation from three experiments analyzed with unpaired *t*-test statistics. (*P < 0.05, **P < 0.01, ***P < 0.001, ****P < 0.0001).

In certain cell types, KSHV primary infection defaults to entering the lytic cycle without establishing latency, such as in lymphatic endothelial cells (LEC), human gingival epithelial (HGEP) cells, and telomerase-immortalized gingival keratinocytes (TIGK) (44–46). To study the role of INTS11 in this non-canonical primary infection route and maintain consistency on the cell type, we explored primary infection using iSLK.RTA cell lines as previously described (29). Distinct from the SLK cells used in the experiments of Fig. 3, we used iSLK.RTA cells and supplemented the cells with Dox upon KSHV infection. In this manner, RTA expression forces KSHV to enter the lytic cycle directly after primary infection. siRNAs were transfected into iSLK.RTA cells before KSHV infection, and the status of KSHV infection was evaluated (Fig. 4A). INTS11 knockdown efficiency and function disruption were validated at the mRNA level (Fig. 4B and C). Upon INTS11 knockdown, the mRNA levels of KSHV lytic genes ORF57, ORF39, and K8.1 were significantly lower than in siNS-treat groups (Fig. 4D through F). KSHV genome copies increase from 8 h to 48 h, suggesting an active lytic replication of viral genomes (Fig. 4G). Significantly fewer viral genome copies were detected when INTS11 was knocked down, suggesting a defective lytic replication without sufficient INTS11 (Fig. 4G). The levels of lytic proteins ORF57 and ORF45 were also repressed when INTS11 was knocked down (Fig. 4H). We also performed similar experiments using a more physiologically relevant model, telomerase-immortalized gingival keratinocytes (TIGK). This cell line has been reported to enter the lytic cycle upon KSHV primary infection (45). As shown in Fig. S3, knockdown of INTS11 also decreased genome copy numbers of KSHV in TIGK, compared with siNS-treated cells. These data suggest that INTS11 plays a critical role in the non-canonical primary infection route, which triggers a spontaneous lytic reactivation instead of establishing latency, a critical phase for viral dissemination from the original infection site. Collectively, INTS11 is essential for optimal lytic replication of KSHV, either directly from primary infection or reactivation from the latent phase.

### INTS9 and INTS6 are required for successful KSHV lytic replication

**Fig 5:**
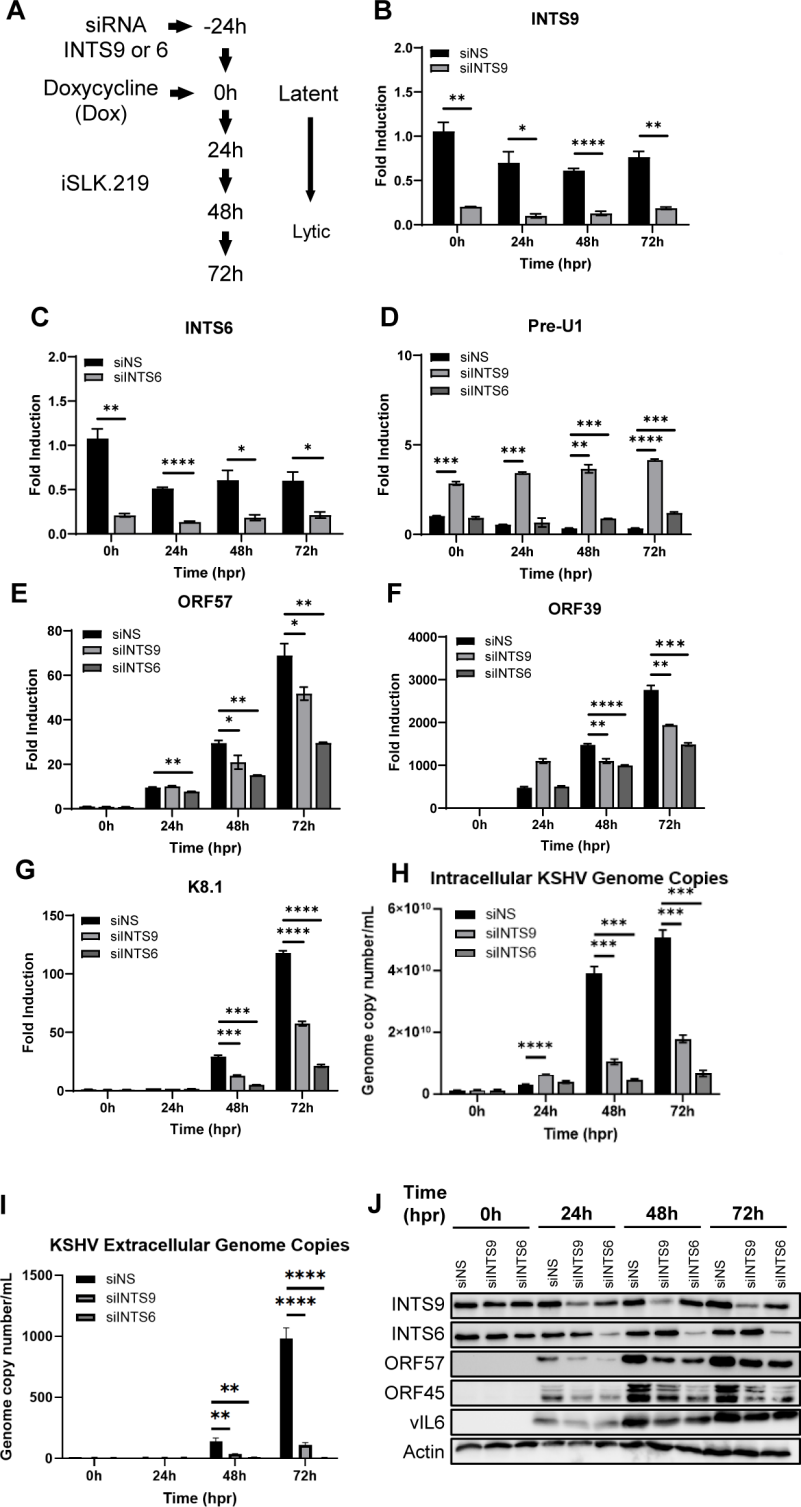
INTS9 and INTS6 facilitate KSHV lytic replication. (**A**) iSLK.219 cells were transfected with a control non-specific (NS) siRNA, siINTS9, or siINTS6 siRNA for 48 h and then treated with doxycycline (Dox, 0.2 µg/mL). (**B–G**) RNA was extracted, cDNA was synthesized, and the knockdown efficiency of (**A**) INTS9, (**B**) INTS6, and the mRNA expression levels of (**C**) Pre-U1 and the KSHV (**E**) ORF57, (**F**) ORF39, and (**G**) K8.1 were measured by real-time PCR. Target mRNA expression was normalized to GAPDH mRNA and presented as fold induction. (**H**) Genomic DNA was extracted and measured for the number of KSHV ORF39 copies/mL with real-time PCR. (**I**) The supernatants were harvested and measured for the number of extracellular KSHV ORF39 copies/mL with real-time PCR. (**J**) Cell lysates were collected at the indicated time points, and western blots were performed with the indicated antibodies. hpr = hours post-reactivation. Error bars are representative of the standard deviation from three experiments analyzed with unpaired *t*-test statistics. (*P < 0.05, **P < 0.01, ***P < 0.001, ****P < 0.0001).

The human INT contains at least 15 subunits, but its molecular mechanism of action is still poorly understood (3, 7, 47). In addition to INTS11, the enzymatic core, INTS9 and INTS6 are relatively better described. INTS9 binds to INTS11 and forms the C-terminal domains of the INT complex, which has been demonstrated to be crucial for the enzymatic function (6, 48). INTS6, coupled with PP2A, is a component of the phosphatase module located on the opposite end of the INT complex (12, 49). This module was shown to play an important role in PP2A-dependent transcription termination. Interestingly, the module is critical at only a subset of integrator-regulated genes, suggesting a selective mechanism that has not been fully explored. Thus, we are curious if INTS9 and INTS6 are also required during the KSHV lytic cycle, and if their function is dependent on INTS11 (Fig. S4). We utilized siRNA to knock down INTS9 or INTS6 in the iSLK.219 lytic reactivation system as shown in Fig. 5A. INTS9 and INTS6 knockdown efficiency and functional disruption of Pre-U1 were validated at the mRNA level (Fig. 5B through D). Similar to the results from the INTS11 knockdown experiment, we also detected significantly lower mRNA levels of ORF57, ORF39, and K8.1 in siINTS9- and siINTS6-treated groups, compared with the siNS control groups (Fig. 5E through G). Knockdown of INTS9 or INTS6 also decreased KSHV cellular genome copies, indicating a defective viral DNA replication (Fig. 5H). Not surprisingly, when INTS9 and INTS6 levels are reduced, defects in all these critical steps result in significantly lower virion production, as quantitated by viral genome copies from the supernatant (Fig. 5I). At the protein level, the knockdown of INTS9 and INTS6 was also validated, and both led to attenuated expressions of viral proteins ORF57, ORF45, and vIL6 (Fig. 5J). Our data confirmed the role of additional INT subunits in KSHV lytic replication. Furthermore, we combined the knockdown of INTS11 to either INTS6 or INTS9 and performed similar assays to assess their impacts on KSHV lytic replication. As shown in Fig. S5A through F, knockdown of INTS6 or INTS9 provided additional attenuation on representative lytic gene transcription and translation.

### INTS11 distinctly regulates KSHV and human gene transcription

**Fig 6:**
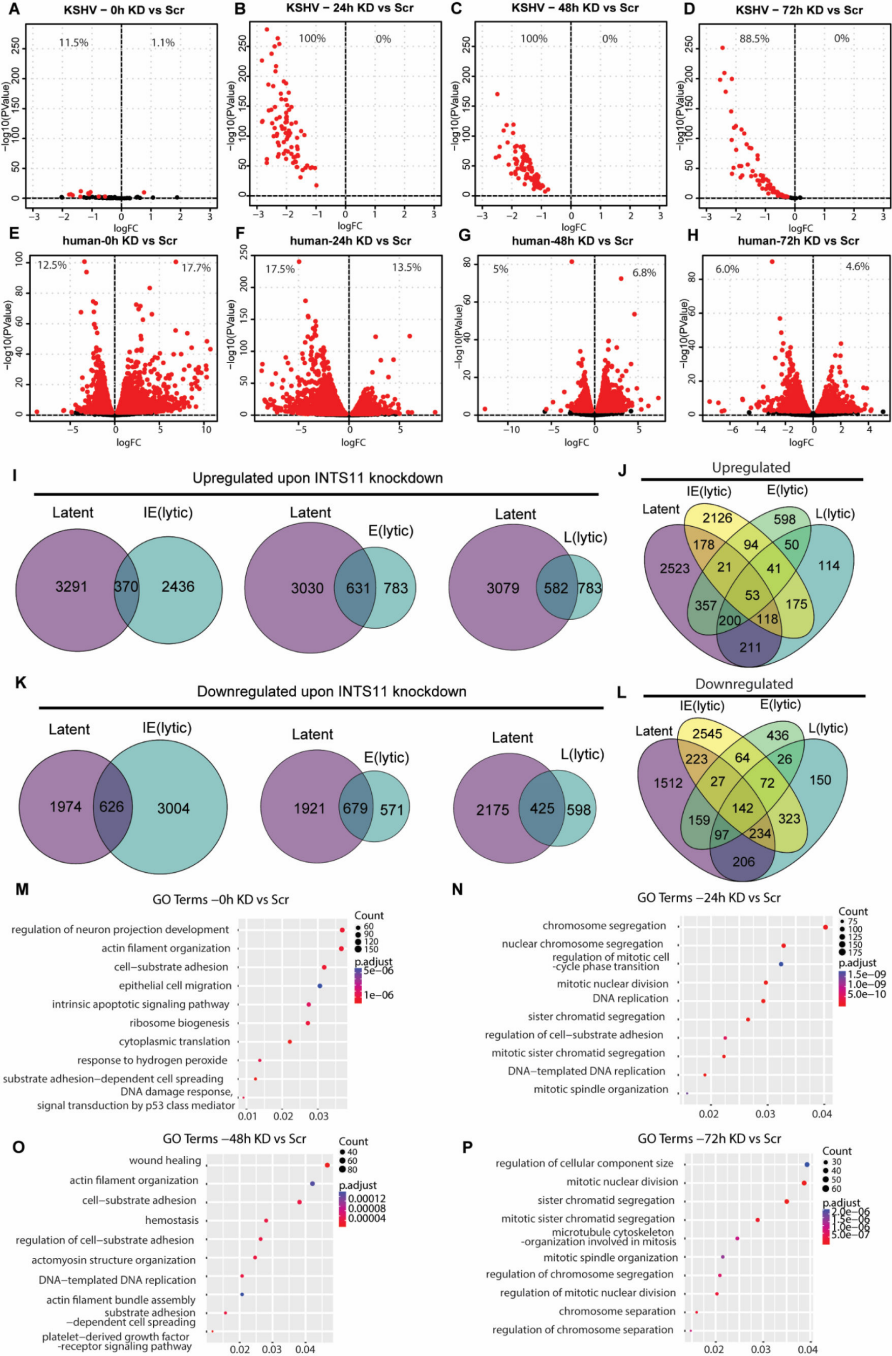
INTS11 distinctly regulates KSHV and human gene transcription. iSLK.219 cells were transfected with control non-specific (NS) siRNA or INTS11 siRNA for 48 h and then treated with doxycycline (Dox, 0.2 µg/mL). RNAs were collected from 0, 24, 48, and 72 h after Dox treatment and subjected to Ribo-depletion RNA-seq. (**A–H**) Volcano plots demonstrate the change in gene expression for INTS11 knockdown cells compared with control cells at latency and 24, 48, and 72 hpr for (**A–D**) KSHV genes and (**E–H**) human genes. Genes exhibiting significantly different gene expression are marked in red. (**I–L**) Venn diagrams comparing the number of differentially expressed human genes at latency and immediate early (IE, 24 h), early (E, 48 h), and late (L, 72 h) lytic time points for (**I and J**) upregulated genes and (**K and L**) downregulated genes. (**M–P**) GO analysis demonstrates the biological processes most enriched in differentially expressed genes upon INTS11 knockdown. hpr = hours post-reactivation.

To investigate the global alteration in the KSHV transcriptome under INTS11 depletion, we performed ribosomal RNA depletion RNA sequencing on iSLK.219 cells subjected to the same conditions as described in Fig. 1B. INTS11-depleted samples were compared with negative control samples at latency (0 h) or different IE, E, and L stages of the lytic cycle at 24, 48, and 72 h. During latency, 11.5% of total KSHV genes’ transcription was negatively impacted by INTS11 deficiency, and 1.1% was upregulated (Fig. 6A). However, after lytic reactivation, 100% (IE), 100% (E), and 88.5% (L) KSHV genes were repressed upon INTS11 knockdown, and none of the lytic genes were upregulated at any lytic stage (Fig. 6B through D). This revealed the significant dependence of the KSHV lytic transcriptome on INTS11, consistent with our KSHV RT-qPCR transcriptome array analysis in Fig. 1J. In contrast to viral genes, only selective sets of human genes were either upregulated or downregulated by INTS11 under each condition, which agrees with the previous reports on INTS11’s role in the human transcriptome without viral infection (6, 50). Moreover, with the progression from the latent to the late lytic phase, INTS11-dependent human genes dropped significantly. Upregulated genes dropped from 12.5% to 6.0% and downregulated genes dropped from 17.7% to 4.6%, showing that a smaller number of human genes were regulated by INTS11 during the late lytic phase than in latency (Fig. 6E through H). This can also be visualized by the Venn diagrams comparing each lytic stage with latency. A decreasing number of genes was observed to be differentially expressed, especially during the early or late stages of the lytic phase, regardless of whether they were upregulated or downregulated (Fig. 6I and K). In addition, there were unique INTS11-dependent genes under each stage, although certain genes remained or switched among different stages (Fig. 6J and L). This suggests a dynamic and complex gene regulation network relying on INTS11 and KSHV infection. GO-term analysis at each stage was performed comparing INTS11 knockdown with WT, and the most significantly altered pathways were listed (Fig. 6M through P).

### Dynamics of INTS11-dependent human gene expression in lytic stages upon reactivation from the latent stage

**Fig 7:**
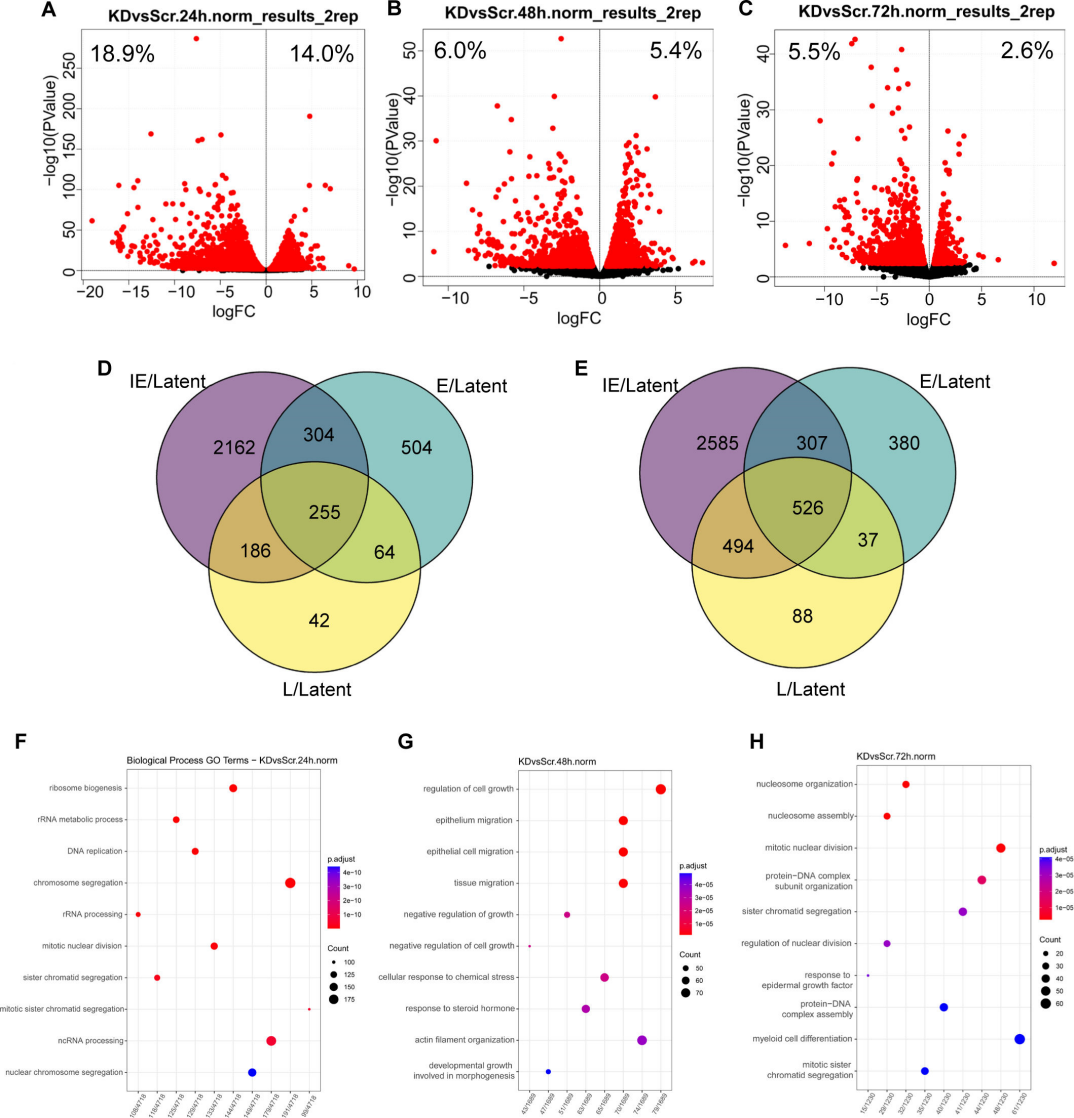
Dynamics of INTS11-dependent human gene expression in lytic stages upon reactivation from latency. iSLK.219 cells were transfected with control non-specific (NS) siRNA or INTS11 siRNA for 48 h and then treated with doxycycline (Dox, 0.2 µg/mL). RNAs were collected from 0 h, 24 h, 48 h, and 72 h after Dox treatment and subjected to Ribo-depletion RNA-seq. (**A–C**) Volcano plots demonstrating human genes that responded differently to INTS11 knockdown at (**A**) 24 h, (**B**) 48 h, and (**C**) 72 h post-reactivation compared with INTS11 knockdown during latency. Genes exhibiting significantly different gene expression are marked in red. (**D and E**) Venn diagrams demonstrate the overlap of genes that are (**D**) upregulated and (**E**) downregulated at immediate early (IE, 24 h), early (E, 48 h), and late (L, 72 h) lytic time points relative to the latent expression. (**F–H**) GO analysis demonstrates the biological processes most enriched in differentially expressed genes upon INTS11 knockdown.

We next focused on dissecting the dependence of human genes on INTS11 when switching from latency to each KSHV lytic stage. Volcano plots were generated using the following formula: [(24 h siINTS11/24 h siNS)/(0 h siINTS11/0 h siNS)]. If a gene ratio of siINTS11/siNS at 24 h (IE) is less than the ratio at 0 h (Latent), it can be concluded that INTS11 plays a weaker role (less dependent) at 24 h (IE) and vice versa. At IE, E, and L stages of the lytic KSHV cycle, 18.9%, 6.0%, and 5.5% of human genes showed weaker INTS11 dependency, whereas 14.0%, 5.4%, and 2.6% showed stronger INTS11 dependency, respectively (Fig. 7A through C). The Venn diagram revealed additional details about the overlap within each comparison. For example, 255 human genes consistently lost INTS11 dependency once KSHV entered the lytic phase, whereas INTS11 exhibited stronger regulatory ability on 526 genes throughout the KSHV lytic cycle (Fig. 7D and E). Moreover, there were many unique or shared genes across different lytic stages, reflecting a dynamic and complex INTS11-dependent RNA metabolism (Fig. 7D and E). We also performed the GO-term analysis based on the dependency changes of INTS11, and the most significantly altered pathways were listed (Fig. 7F through H).

### INTS11 is recruited to the KSHV genome during the lytic cycle

**Fig 8:**
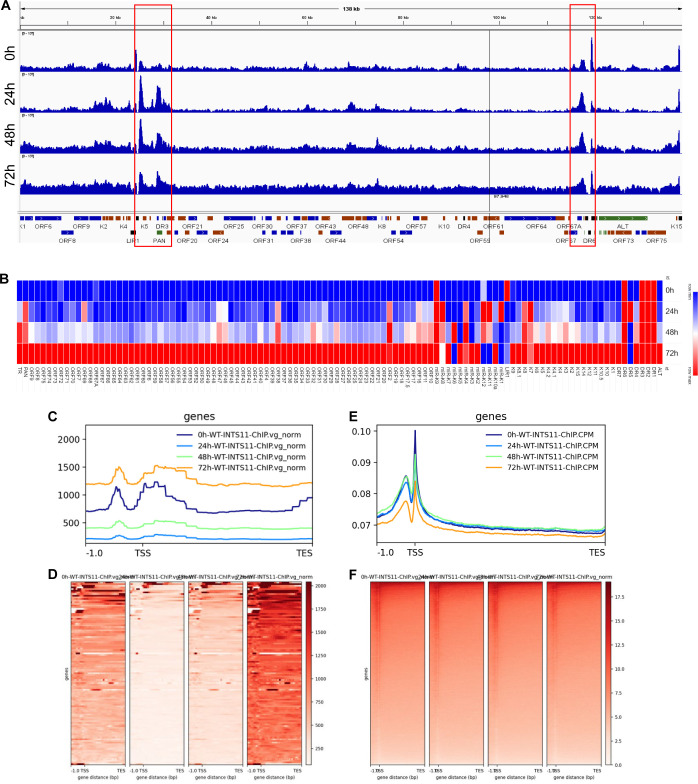
ChIP-seq data reveal the preferential binding sites of INTS11 on the KSHV genome. iSLK.219 cells were transfected with control non-specific (NS) siRNA or INTS11 siRNA for 48 h and then treated with doxycycline (Dox, 0.2 µg/mL). IgG served as a control for normalization. (**A**) Coverage of INTS11 ChIP-seq reads across the KSHV genome. (B) Coverage of INTS11 ChIP-seq reads across the KSHV ORFs. (**C and E**) Metagene plots show the average binding position of INTS11 across all genes in the (**C**) KSHV and (**E**) human genomes. (**D and F**) Heatmaps showing the abundance of INTS11 reads across (**D**) KSHV and (**F**) human genes. All panels (**A–F**) use INTS11 read abundance normalized to the abundance of KSHV viral genomes in the ChIP input samples.

To define the activity of the Integrator complex during KSHV reactivation from latency, we generated chromatin immunoprecipitation sequencing profiles during latency and different lytic stages. ChIP-seq analysis revealed two INTS11 recruitment hotspots on the viral genome (Fig. 8A). The left hotspot spanned from position ~23–32 kb and covered the left origin of KSHV lytic replication (Ori-L), the long (∼1 kb) inverted repeat (LIR1), various KSHV ORFs (K4, K4.1, K4.2, K5, K6, and ORF16), and long non-coding RNA PAN. The right hotspot overlapped with ORF69, K12, Kaposin, and the right origin of lytic replication (Ori-R) (Fig. 8A, position ∼117–120 kb). Metagene analyses of the ChIP-seq read showed that within all groups, INTS11 preferably bound to the transcription start site (TSS) surrounding regions of all KSHV ORFs, consistent with previously reported INTS11 recruitment preference on human genes (51, 52). In addition, we observed increasing total INTS11 ChIP-seq reads as KSHV reactivated from latency and progressed to the late lytic phase (Fig. 8B). Consistently, we observed an increasing density of INTS11 binding without binding site preference alteration within KSHV ORFs (Fig. 8D). We next evaluated if INTS11 would be recruited to the human genome differently under latent and lytic viral infection. No significant binding site preferences among each group were seen, as INTS11 aggregated to the same TSS regions under all conditions (Fig. 8C). However, there was a decrease in INTS11 recruitment to the human genome at 72 h after KSHV entered its lytic cycle, in contrast with the increased detection of INTS11 on the KSHV genome (Fig. 8C). This is also reflected in Fig. 8E, where the intensity of INTS11 reads was dropped at the KSHV late lytic stage, without altering its binding patterns. Collectively, our data suggest that INTS11 is hijacked to the KSHV genome during KSHV lytic reactivation, but the binding site preference is unchanged.

## DISCUSSION

RNA metabolism is essential for the homeostasis of all living organisms and is always adapted to perturbation. Therefore, it is crucial to understand the biological functions of INT-mediated RNA metabolism in a dynamic system, not merely at the baseline state. Viruses have evolved to take over the cell to produce viral progeny, hijacking cellular RNA metabolic machinery for viral benefit, thereby providing an ideal dynamic system to interrogate transcriptional regulations. In this study, we report that INT plays a critical and global role in regulating the viral transcriptome, and the virus hijacks the Integrator complex to facilitate its viral life cycle, concurrently limiting the accessibility of the Integrator complex to the human genome.

Multiple studies have revealed the fundamental roles of the integrator complex in RNA metabolism. Previous studies of INTS11’s role in regulating human gene transcription reported that the knockdown of INTS11 both positively and negatively impacts subsets of genes, instead of causing global repression of the host transcriptome (1, 3, 6, 50). However, in our iSLK.219-based KSHV reactivation model, INTS11 plays a pivotal role in facilitating all viral genes during the lytic stage of KSHV. This phenotype was validated using an RT-qPCR-based KSHV array as well as RNA-sequencing, suggesting that INTS11 is preferentially recruited to the viral genome during lytic replication.

Given the profound impact of INTS11 on the lytic phase of KSHV in the lytic reactivation model, we asked if it also facilitated other phases of the KSHV life cycle. Thus, we used two different models to study primary infection following infectious virion production, the default primary infection route in most of the cells (latency), and the non-canonical primary infection route (lytic). In the default primary infection route, we did not observe a clear impact on KSHV infection after removing INTS11 because comparable numbers of KSHV genomes were detected in both siNS and siINTS11 groups. Therefore, INTS11 plays a minimal role in viral entry and establishing latency in host cells. However, the loss of INTS11 significantly attenuated representative KSHV lytic gene transcription levels in the non-canonical route model (lytic), suggesting that INTS11 preferentially affects viral gene transcription during the lytic phase. Interestingly, for a short period of time, we did not observe significant repression of latent genes when the INTS11 level is low in iSLK.219 cells. This is also limited by the siRNA technology. Unfortunately, our attempts to generate a CRISPR-based knockout or shRNA-based knockdown of INTS11 in iSLK.219 or iSLK.BAC16 failed multiple times. This is possibly due to the previous report that repression of INTS11 will lead to G1-S arrest and eventually cell death (53), leaving a technical challenge to investigate the long-term impact of the INT complex to KSHV latent maintenance.

There are 15 identified integrator subunits, which intrigues us to study the role of other subunits in KSHV lytic replication (3, 7, 47). We also characterized INTS9, which forms the enzymatic core of INT with INTS11 (48), and INTS6, which has been shown to recruit PP2A for some INTS11-independent functions (12, 49). Similar to INTS11, both INTS9 and INTS6 facilitated KSHV viral transcription, translation, genome replication, and infectious virion production, suggesting that the integrator complex is critical for KSHV lytic replication. Notably, INTS6 knockdown did not result in elevated pre-U1 accumulation until late stages (48 h and 72 h) of the KSHV lytic phase. It is possible that phosphatase recruitment is less important for INT function compared with the endonuclease activity, facilitated by INTS9 and INTS11. This could reflect the relatively weaker influence of INTS6 on U1 processing, which requires a longer accumulation period. The combined siRNA experiments showed that either loss of INTS6 or INTS9 with INTS11 further repressed KSHV lytic replication, suggesting that INTS6 or 9 has additional functions that are independent of INTS11. However, due to the limitations of siRNA technology, we cannot eliminate INTS11 in our cells. Thus, we cannot rule out the possibility that the further inhibition of KSHV lytic gene transcription or translation is due to INTS6/9’s loss in an INTS11-dependent manner. With the challenge of INTS11 KO cell generation, the detailed mechanisms of each subunit during KSHV lytic replication are still open questions.

The RNA-seq analyses revealed distinct roles of INTS11 in viral and host RNA metabolism. Compared with the bi-directional and proportional INTS11-dependent regulation, KSHV lytic genes rely on INTS11 for optimal lytic replication. More interestingly, with the progression of the lytic cycle, we observed a clear declining trend in the INTS11-dependent host gene population, suggesting that a lower number of human genes were regulated by INTS11 during the lytic phase compared with the latent phase. The INTS11-dependent genes were further categorized and visualized by Venn diagrams based on KSHV life cycle stages. There are unique and overlapping genes at different stages, which are worth further exploring to dissect INTS11’s role in host RNA metabolism. Looking from a different angle, in addition to gene populations, we also revealed that host gene interaction with INTS11 was attenuated as the lytic phase progressed. The composition of unique or overlapped genes was also categorized. In the end, we have also analyzed the most affected biological processes under each condition. The RNA-seq-based analysis revealed affected pathways and genes, suggesting a dynamic network of RNA metabolism and unknown mechanisms to be explored.

Close examination of the INTS11 enrichment sites from the ChIP-seq data revealed two specific hotspots on the viral genome during KSHV lytic reactivation. One of them covers polyadenylated nuclear (PAN) RNA, a long non-coding RNA that is highly expressed in the immediate early phase and plays a crucial role in facilitating KSHV lytic reactivation (54, 55). It was reported that RTA binding to the PAN promoter leads to the recruitment of RNAPII, which is subsequently trapped by a DNA loop formed by RTA, ORF57, and the PAN region (28). With the previously described association of INT with RNAPII, INTS11 likely affects global KSHV transcriptional changes through involvement with RNAPII, which is recruited and trapped by RTA, ORF57, and PAN RNA, crucial components driving a subsequent robust lytic reactivation.

In all, we have shown that INTS11 is essential for optimal KSHV mRNA metabolism and facilitates optimal lytic replication. INTS11 deficiency caused global repression of the KSHV transcriptome, although its impact on human transcription remains bidirectional. Our data revealed dynamic and complex INTS11-dependent human gene regulations under each stage of the KSHV life cycle. Mechanistically, during the lytic cycle, INTS11 is recruited to the KSHV genome broadly as well as with some specific binding site preference. However, the detailed mechanisms are still open to questions. Our study can also be expanded to assess other viruses, which allows for dissecting INT’s role more widely in host-pathogen interactions.

## Data Availability

The RNA-seq data and ChIP-seq data are available at NCBI Gene Expression Omnibus (GEO) under accession numbers GSE300543 and GSE300544, respectively.

## SUPPLEMENTAL MATERIAL

Fig. S1 jvi.00266-25-s0001.tif INTS11 knockdown inhibits lytic reactivation of KSHV in BCBL1 cells.

Fig. S2 jvi.00266-25-s0002.tif Impact of INTS11 knockdown combination on KSHV latent gene transcription in iSLK.219 cells.

Fig. S3 jvi.00266-25-s0003.tif INTS11 knockdown led to less KSHV infection in telomerase-immortalized gingival keratinocytes (TIGK).

Fig. S4 jvi.00266-25-s0004.tif Simplified demonstration of INTS11/9/6 in the integrator complex.

Fig. S5 jvi.00266-25-s0005.tif Impact of INTS11/6/9 knockdown combination on KSHV lytic reactivation in iSLK.219 cells.

Fig. S6 jvi.00266-25-s0006.tif Failure to generate CRISPR-based INTS11 KO iSLK cell lines.

Table S1 jvi.00266-25-s0007.xlsx Differential expression of human genes.

Table S2 jvi.00266-25-s0008.xlsx KSHV array primers.

ASM does not own the copyrights to Supplemental Material that may be linked to, or accessed through, an article. The authors have granted ASM a non-exclusive, world-wide license to publish the Supplemental Material files. Please contact the corresponding author directly for reuse.
