# Supplementary figures and images for: Essential function of the integrator complex in Kaposi’s sarcoma-associated herpesvirus lytic replication
Source: J Virol. 2025 Aug 13;99(9):e00266-25. doi: 10.1128/jvi.00266-25 (PMC12455965; doi:10.1128/jvi.00266-25)

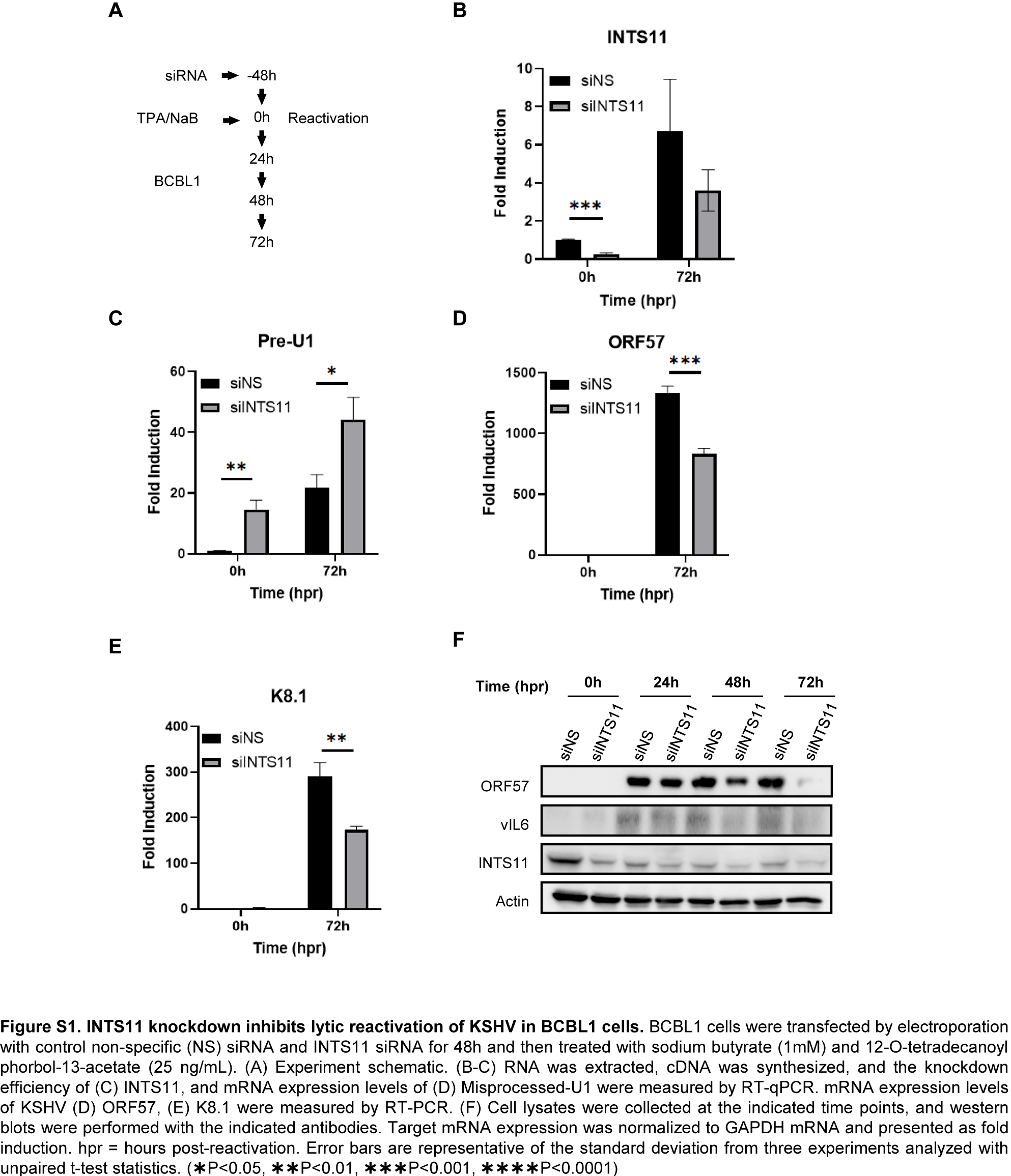

Supplement: Fig. S1 — INTS11 knockdown inhibits lytic reactivation of KSHV in BCBL1 cells. [file jvi.00266-25-s0001.tif]

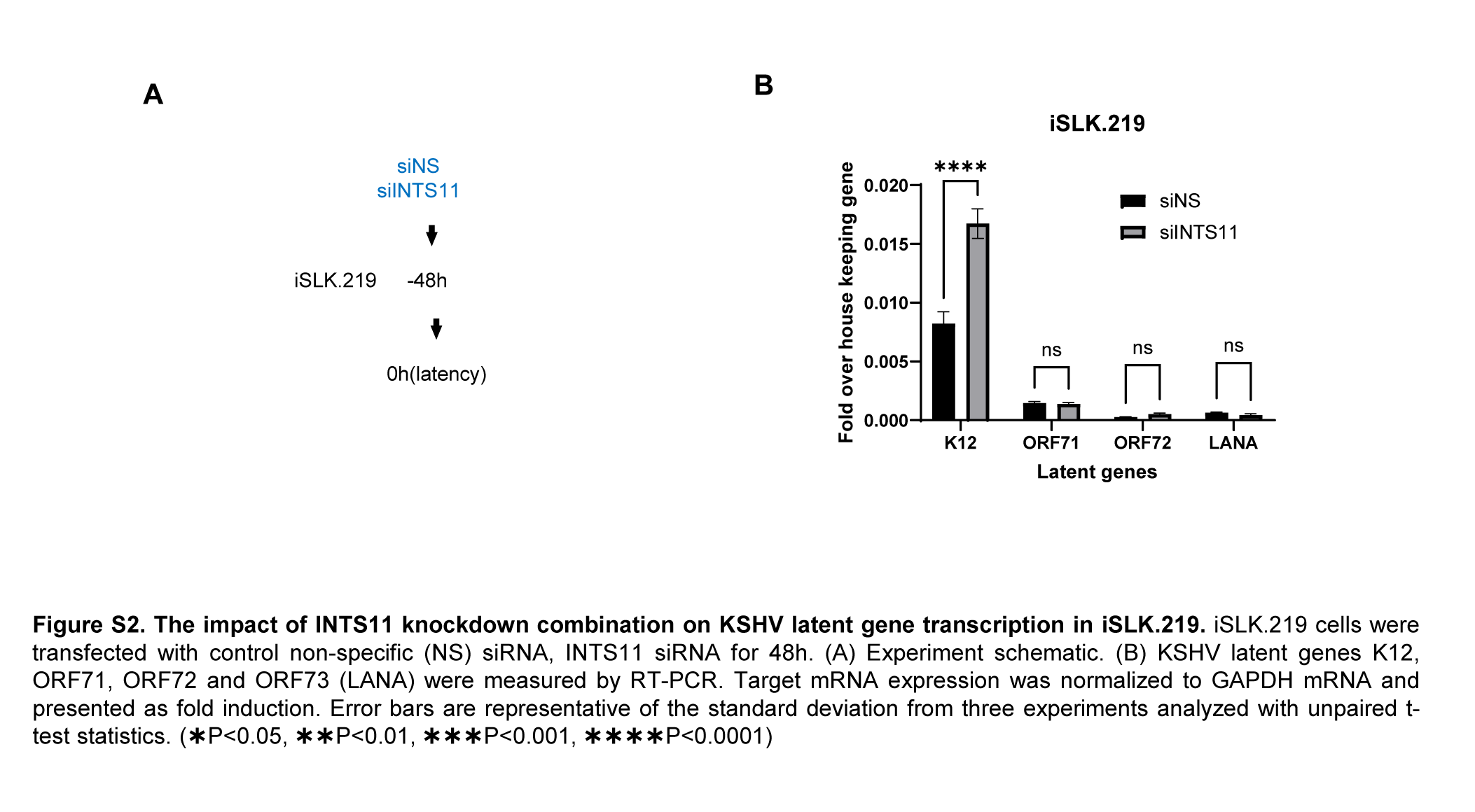

Supplement: Fig. S2 — Impact of INTS11 knockdown combination on KSHV latent gene transcription in iSLK.219 cells. [file jvi.00266-25-s0002.tif]

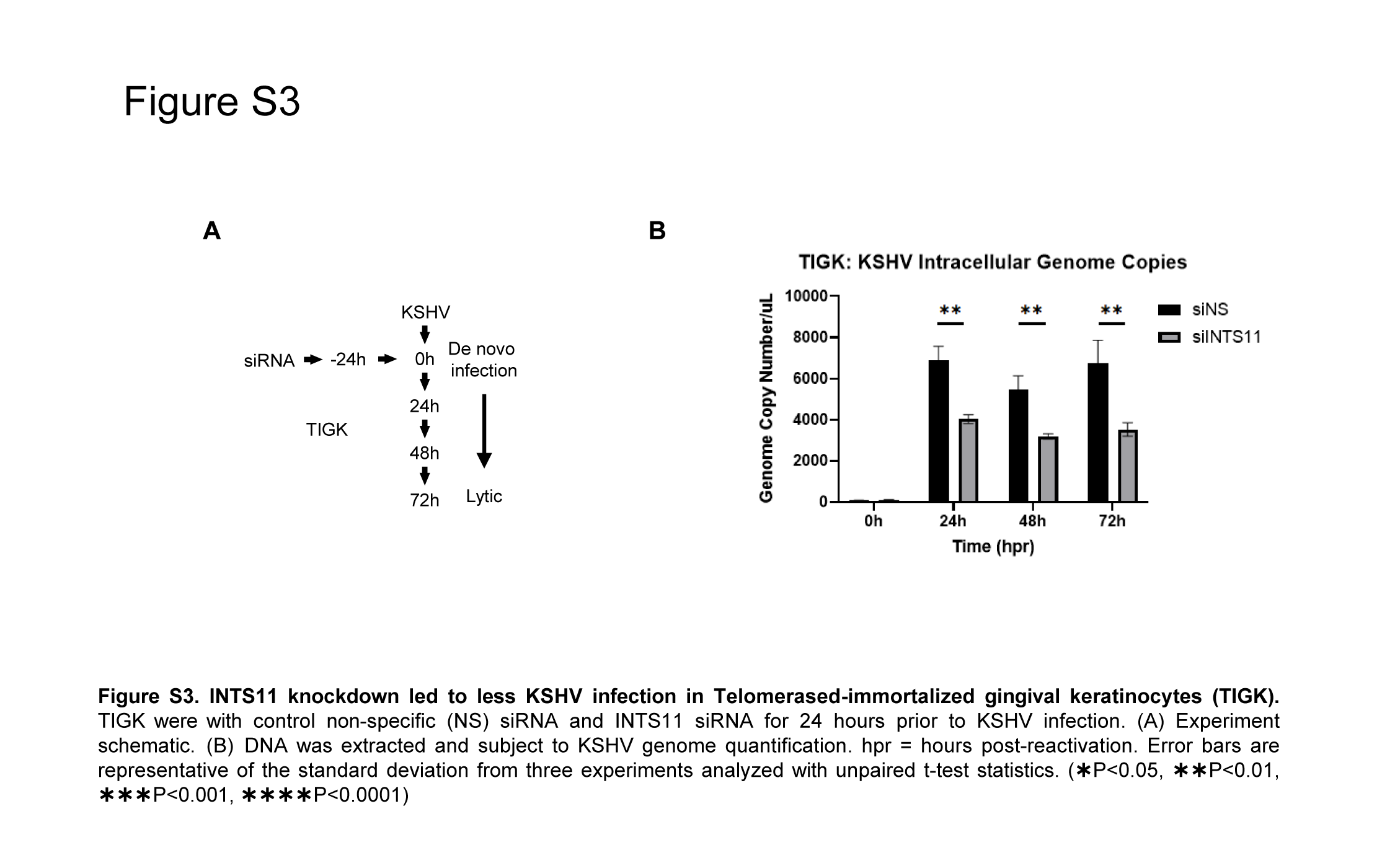

Supplement: Fig. S3 — INTS11 knockdown led to less KSHV infection in telomerase-immortalized gingival keratinocytes (TIGK). [file jvi.00266-25-s0003.tif]

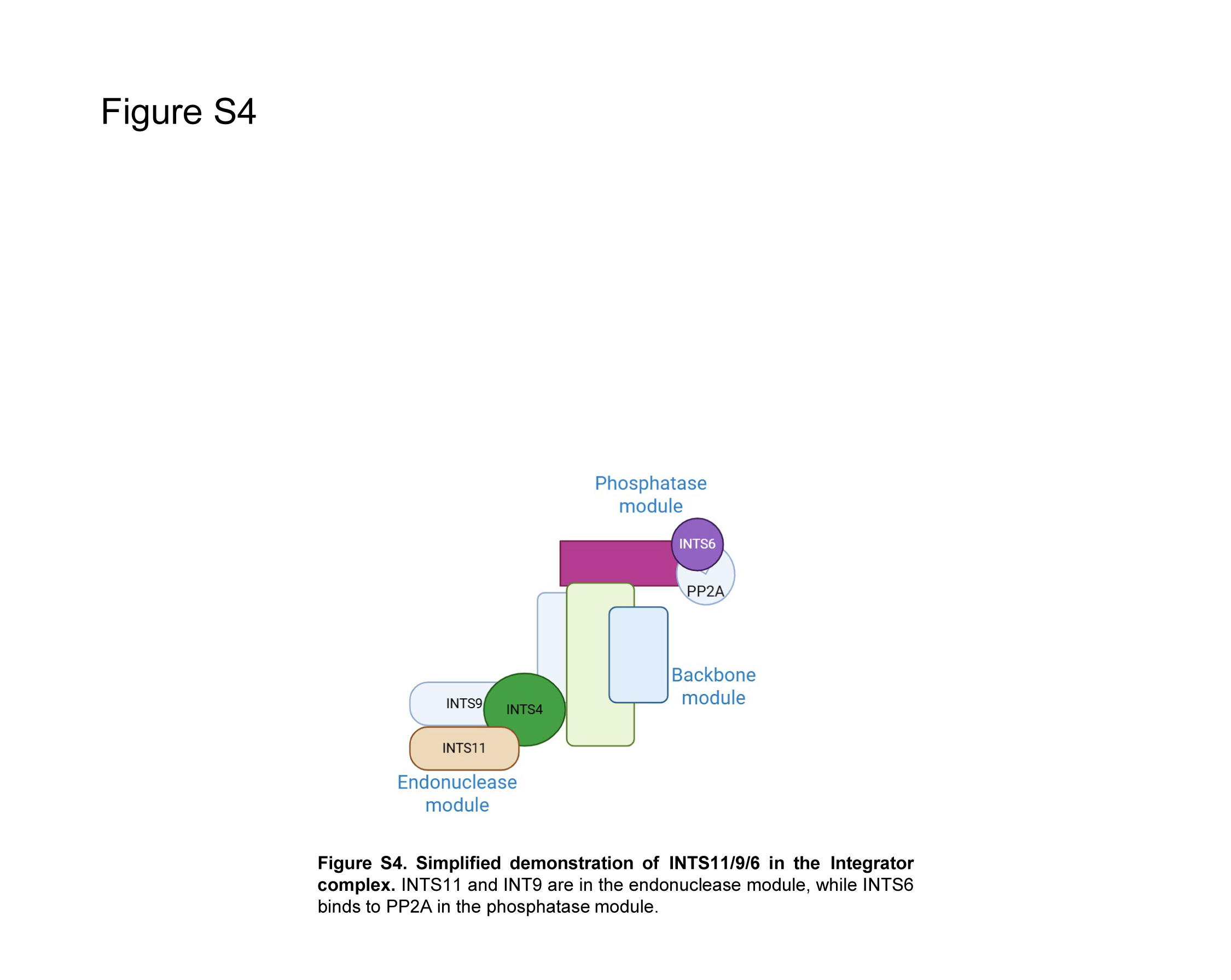

Supplement: Fig. S4 — Simplified demonstration of INTS11/9/6 in the integrator complex. [file jvi.00266-25-s0004.tif]

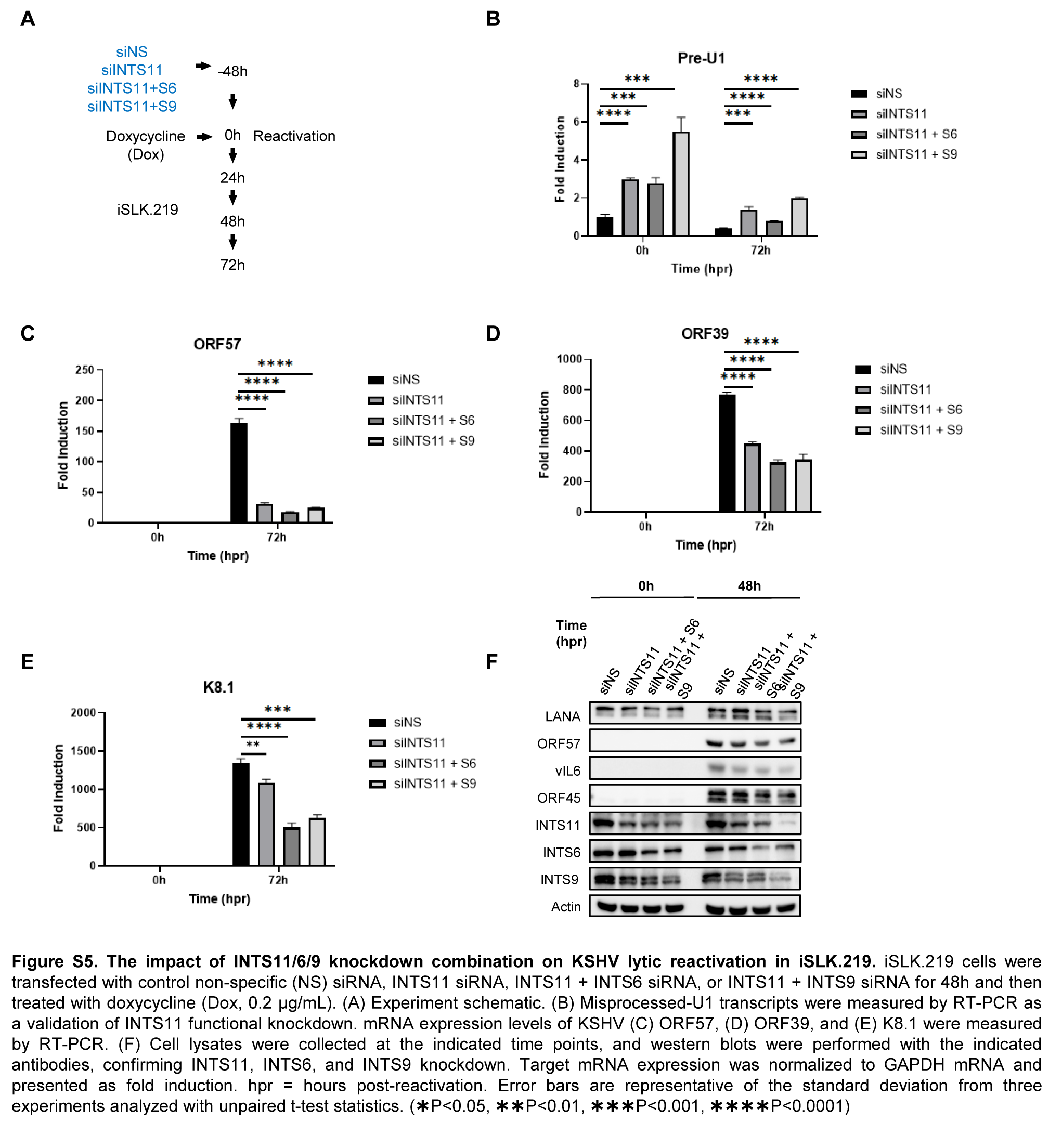

Supplement: Fig. S5 — Impact of INTS11/6/9 knockdown combination on KSHV lytic reactivation in iSLK.219 cells. [file jvi.00266-25-s0005.tif]

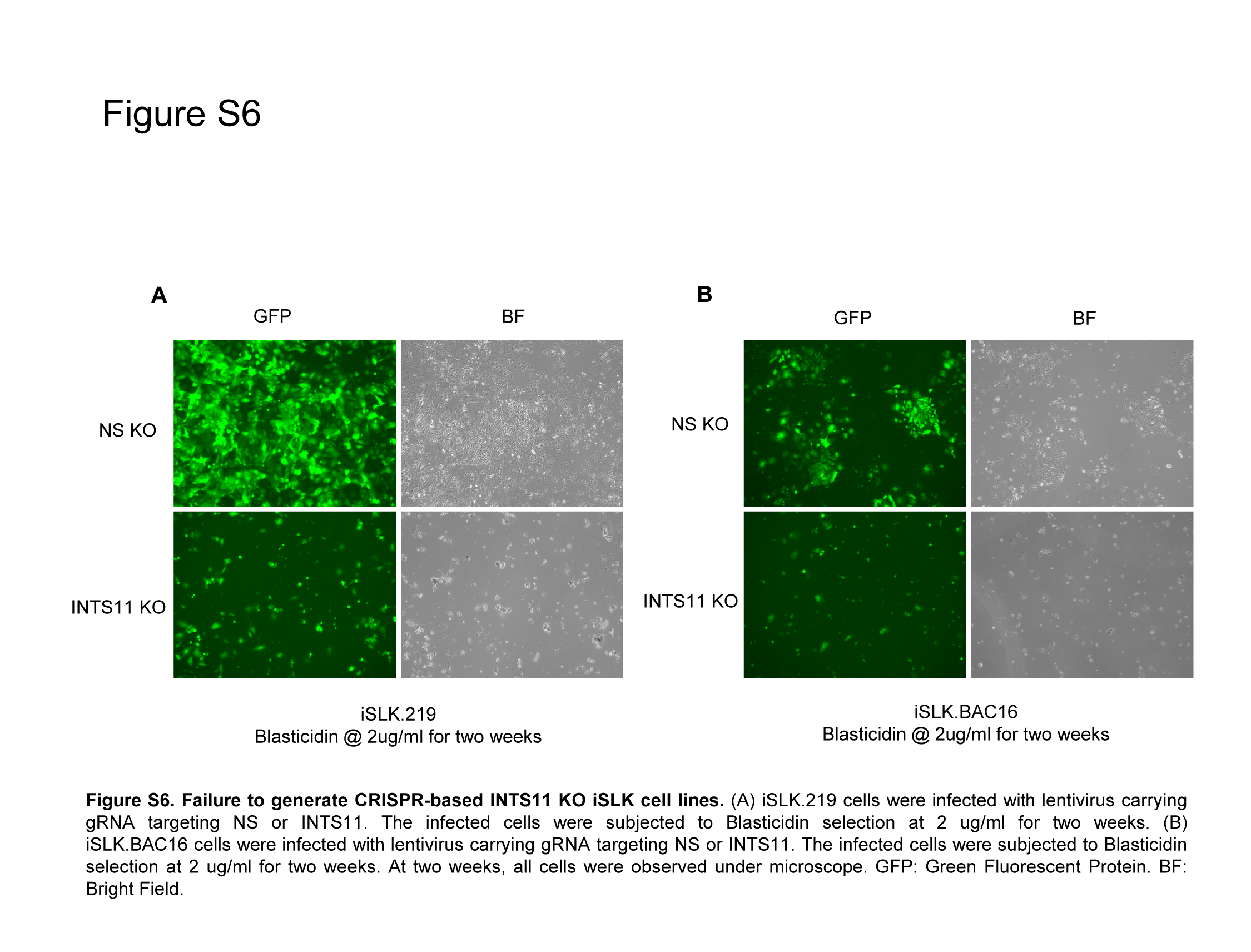

Supplement: Fig. S6 — Failure to generate CRISPR-based INTS11 KO iSLK cell lines. [file jvi.00266-25-s0006.tif]
